# Healthcare professionals' perceptions of patient safety for the woman in childbirth in Sweden – An interview study

**DOI:** 10.1002/nop2.435

**Published:** 2019-12-18

**Authors:** Annika Skoogh, Carina Bååth, Ann‐Kristin Sandin Bojö, Marie Louise Hall‐Lord

**Affiliations:** ^1^ Department of Health Sciences Faculty of Health, Science and Technology Karlstad University Karlstad Sweden; ^2^ Faculty of Health and Welfare Östfold University College Fredrikstad Norway; ^3^ Department of Health Science Faculty of Medicine and Health Sciences Norwegian University of Science and Technology Gjøvik Norway

**Keywords:** healthcare worker, interviews, intrapartum, phenomenography, quality and safety

## Abstract

**Aim:**

To describe healthcare professionals' perceptions of patient safety with a focus on the woman in connection to childbirth.

**Design:**

A descriptive and qualitative design with a phenomenographic approach.

**Methods:**

Individual qualitative face‐to‐face interviews with 19 healthcare professionals (midwives, nursing assistants and physicians) were conducted in three labour wards in Sweden. The data were analysed according to Dahlgren and Fallsberg's seven steps.

**Results:**

The informants' perceptions of patient safety for the women were identified in four qualitative different descriptive categories: *Safeguarding the woman*, *Safeguarding the birth process*, *Respecting the individual and the team* and *Managing workforce and learning*. Supportive care and listening to the woman were important for patient safety. It was crucial to follow labour stages and to avoid unnecessary interventions. An open and tolerant atmosphere between the healthcare professionals improved decision‐making, and a reasonable workload was essential for ensuring safe care.

## 1 INTRODUCTION

The healthcare organizations are complex, and the quality of care and patient safety depends on the healthcare professionals working together in interprofessional teams (Reeves, Lewin, Espin, & Zwarenstein, 2010). Patient safety is highly prioritised, and efforts have been made to reduce healthcare‐associated infections, pressure ulcers and falls (SALAR, 2019; WHO, 2019). In intrapartum care, patient safety is included in the recommendations for a positive childbirth experience (WHO, 2018). Patient safety is defined as “the reduction of risk of unnecessary harm associated with health care to an acceptable minimum” (WHO, 2009, p. 15). Despite healthcare professionals' intentions not to harm, almost ten per cent of hospitalized patients suffer harm from health care with nearly half of these incidents being preventable (de Vries, Ramrattan, Smorenburg, Gouma, & Boermeester, 2008), which constitutes a human and costly challenge (Agency for Healthcare Research & Quality, 2019). Most incidents in patient care occur not from actions by individuals but from systems that are in conflict or not optimal. Furthermore, the organizations may lack consistency in supporting the healthcare professionals to deliver safe care (Carayon et al., 2006). It may be a challenge for healthcare professionals to have knowledge and understand patient safety in their clinical practice. The present study contributes to the further understanding of patient safety in intrapartum care with a focus on the woman from the perspective of front‐line healthcare professionals.

## 2 BACKGROUND

Most births are uneventful for the women involved, even if unexpected complications and patient harm can occasionally occur (Danilack, Nunes, & Phipps, 2015; WHO, 2017). Maternal death due to pregnancy and childbirth is unusual and considered a major human tragedy (Nyfløt, Ellingsen, Yli, Øian, & Vangen, 2018). Research about patient safety in connection to childbirth often targets the neonate (Ashcroft, 2008; Millde Luthander, 2016), with fewer studies focusing on the woman giving birth (Jacobson, Zlatnik, Kennedy, & Lyndon, 2013; Sheldon et al., 2014).

Studies of intrapartum care have found that midwives, physicians and nurses all experience safety concerns (Maxfield, Lyndon, Kennedy, O'Keeffe, & Zlatnik, 2013) and patients being put at risk due to a failure to listen to or respond to safety concerns (Lyndon et al., 2014). Furthermore, nurses perceived a risk of harmful interventions and lack of information to the woman giving birth (Jacobson et al., 2013). Another patient safety problem raised in intrapartum care is communication between nurses and physicians (Lyndon et al., 2012). Inadequate communication is a common contributing factor to patient harm (The Joint Commission, 2017).

A midwife's responsibility in intrapartum care is to protect and improve women's health outcomes and reduce the use of unnecessary interventions (International Confederation of Midwives; Renfrew et al., 2014) such as augmentation of labour, instrumental vaginal birth and caesarean section (WHO, 2018). In Sweden, midwives in labour wards have an autonomous professional responsibility to take care of women going through what is expected to be normal childbirth in collaboration with a nursing assistant. If complications arise during childbirth, midwives will collaborate with physicians (Svenska Barnmorskeförbundet, 2018). Only a few studies on patient safety in intrapartum care concerning women from the perspective of healthcare professionals have been conducted. The aim of the study was, therefore, to describe healthcare professionals' perceptions of patient safety with a focus on the woman in connection to childbirth.

## 3 METHODS

### 3.1 Design

The study had a descriptive and qualitative design with a phenomenographic approach. The purpose of the phenomenographic approach is to distinguish and identify variations in perceptions of a specific phenomenon and to qualitatively describe different ways this could be experienced and understood by a collective group of people, recognized from a “second order perspective,” which explores how people experience or perceive a phenomenon. In contrast, the “first order perspective” is about the phenomenon itself (Marton, 1981). The phenomenon explored in this study is patient safety with a focus on the woman in connection to childbirth.

### 3.2 Settings and informants

The study took place in the labour wards of three mid‐size hospitals in Sweden. All midwives, nursing assistants and physicians working in the wards were invited to participate. They were informed orally and in writing about the study and were invited to contact the first author if they were interested in participating in the study. When using a phenomenographic approach, purposive sampling is important to achieve variation in informant characteristics (Marton, 1981). In this study, the intention was to obtain variation in age and work experience in intrapartum care. In total, 20 healthcare professionals responded that they were interested in participating. They were contacted by the first author, and 19 consented to participation. The characteristics of the informants are described in Table 1.

**Table 1:** Characteristics of informants (*n* = 19)

	*n*
Healthcare professionals	
Midwife	8
Nursing assistant	5
Physician	6
Sex	
Female	15
Male	4
Age groups (years)	
26–35	1
36–45	5
46–55	4
≥56	9
Experiences in intrapartum care (years)	
≤10	6
11–35	7
≥36	6

### 3.3 Data collection

Individual qualitative face‐to‐face interviews were conducted by the first author during the period August 2016–June 2017. All the interviews took place in the hospitals informants worked in, with the exception of one, where the informant preferred to be interviewed at home. Two interview questions were asked. *What does patient safety with a focus on the woman in childbirth mean to you?* and *How do you perceive patient safety with a focus on the woman in childbirth, based on your experience?* Further probe questions were asked such as *Can you express yourself further? Can you give an example? Could it vary?*

Two pilot interviews were conducted by the first author to practice interview techniques and test the interview questions (Bowden & Green, 2005). The pilot interviews were included in the study as no changes to questions were needed. The interviews were digitally recorded and lasted between 36 and 94 min (median 54 min). The interviews were transcribed verbatim.

### 3.4 Data analysis

Data were analysed according to Dahlgren and Fallsberg's (1991) seven steps. Analysis was performed by the first author in close collaboration with the other authors. In the first step, *Familiarization*, the transcripts were read several times to get familiar with the whole content and to establish an overall impression of the data. In the second step, *Condensation*, significant statements made by the informants were selected. The statements were condensed to get a short but representative version of the entire dialogue concerning the phenomenon. In the third step, *Comparison*, a comparison of the selected significant statements was made to find sources of variation or agreement. In the fourth step, *Grouping,* answers which appeared to be similar were put together. In the fifth step, *Articulating*, a preliminary attempt to describe the essence of similarity in each group of answers was obtained. In the sixth step, *Labelling*, various perceptions were denoted by constructing a suitable linguistic expression. In the last step, *Contrasting,* the obtained perceptions were compared for similarities and differences. Throughout the whole analysis, the authors went back and forth between the steps and the whole (Dahlgren & Fallsberg, 1991). The outcome space is presented in a horizontal view, containing descriptive categories and perceptions (Uljens, 1996).

### 3.5 Rigour

To establish overall trustworthiness, we used Lincoln and Gubas' (1985) four criteria to be met; credibility, confirmability, dependability and transferability. *Credibility* was strengthened during data collection and analyses process with different techniques. Open‐ended questions encouraged the informants to talk openly about their perceptions. The first author's (AS) understanding of the data in all steps was repeatedly discussed with the other authors (CB, AKSB, MLHL) during the whole analysis process, for keeping attention to the “second order perspective” of the phenomenon being studied. The assessment of c*onfirmability* was traced back by the “audit trail,” bringing an objective perspective to the phenomenon. *Dependability *was secured by using the same two open‐ended questions in all interviews and by using excerpts to support the relation to the perception. *Transferability* was achieved through rich “thick descriptions” in the data and informants recruited from three different geographic settings, and therefore, the findings may be transferable in a Swedish context.

### 3.6 Ethics

The study was approved by the research ethics committee at Karlstad University (C2016/363). The researchers followed the ethical guidelines (WMA Declaration of Helsinki, 2013) concerning confidentiality integrity and voluntariness. The study was approved by the head of the department at the labour wards.

## 4 RESULTS

The analysis was identified in four descriptive categories containing nine perceptions, generated from the informants' perceptions of patient safety with a focus on the woman in connection to childbirth (Table 2).

**Table 2:** The outcome space with descriptive categories and perceptions

Descriptive categories	Safeguarding the woman	Safeguarding the birth process	Respecting the individual and the team	Managing workforce and learning
Perceptions	Supporting the woman	Following the stages of labour	Using each other's competence	Having a reasonable workload
	Listening to the woman	Avoiding unnecessary interventions	Striving for openness and a tolerant atmosphere	Learning from critical incidents
			Supporting new colleagues	

### 4.1 Safeguarding the woman

The descriptive category includes two perceptions: *Supporting the woman* and *Listening to the woman*.

#### 4.1.1 Supporting the woman

Supporting the woman describes the importance of a midwife or nursing assistant being present with the woman in the labour room to create safe conditions. Being present is connected with a lower frequency of tearing and less need for pain relief and augmentation of labour. Continuity in care was described to be vital in creating a relationship “…sometimes I feel that it went so well because I had been there the whole time.” The presence of a midwife or nursing assistant in the labour room lead to lower levels of anxiety and physical tension in the woman “…I kept my hand on her [shoulder] and we spoke quietly about what she was feeling.” It was also described that being continuously present is not ultimately necessary, as it is more important that the woman receives the best medical treatment available. Supporting the woman during labour also includes offering fluids and food, helping her to change position and motivating her to get up and move about.

#### 4.1.2 Listening to the woman

The informants described the importance of having as complete picture as possible of the woman's situation during childbirth. When a woman shares information, her care becomes safer, and the risk of unnecessary interventions and potential patient harm is reduced. Being open and listening to the woman's feelings, needs and desires was described as being meaningful. Providing information to the woman about the various alternatives available during childbirth was stated as important “…she would have preferred to have it presented to her along with the alternatives … perform an instrumental vaginal birth or …. an emergency caesarean section.”

Others described that the woman should not always be part of medical decisions and that there are situations where it is not safe to grant her what she wants, for example to perform an emergency caesarean section or to induce labour.

If the woman and the healthcare professionals do not speak the same language, warning signs may not be disclosed, and risky situations can develop due to the woman not being able to communicate. This can also be the case when information is missing in a woman's birth records.

### 4.2 Safeguarding the birth process

This descriptive category consists of two perceptions: *Following the stages of labour* and *Avoiding unnecessary interventions*.

#### 4.2.1 Following the stages of labour

Observing, reporting and monitoring of the woman during childbirth was said to be crucial for patient safety. The informants described the importance of having knowledge of risk conditions such as obesity and complications that can occur during childbirth, which may lead to a negative birth experience. Using a structured communication method at shift changes and during rounds was described as reducing the risk of missing information. “Before we started using this method … we often got so much information that we sometimes didn't know what to do with it, everything just merged into one. The more information you get, the less you can absorb and then it is easy to miss important information.”

Another approach taken to strengthen patient safety is to involve both the woman and her partner when performing a handover in the labour room. Easily accessed guidelines were described to support monitoring and follow‐ups during childbirth. Unintended poor monitoring after childbirth may result in a postpartum haemorrhage that could lead to blood transfusions and prolonged hospital stays.

#### 4.2.2 Avoiding unnecessary interventions

Interventions such as the induction of labour, caesarean section and instrumental vaginal birth can affect patient safety and lead to potential patient harm “…in some cases it [instrumental vaginal birth] is used on women whose contractions are weak where actually all that is needed is a little more patience or a higher infusion dose [augmentation of labour].”

Other informants described the value of a “time‐out” where healthcare professionals discuss the necessity of instrumental vaginal birth. Another statement made was that interventions to strengthen labour are necessary when labour is not progressing.

### 4.3 Respecting the individual and the team

The descriptive category includes three perceptions; *Using each other's competence*, *Striving for openness and a tolerant atmosphere* and *Supporting new colleagues*.

#### 4.3.1 **Using each other**'**s competence**

The informants described the importance of knowing each other's competence and role, especially in emergencies when many actions are performed simultaneously “… we are familiar with each other's competence and can complement and help each other.” If healthcare professionals are not familiar with each other's competence, uncertainty and lack of trust can develop. Team training was described to lead to a better understanding of each other's competence and knowing what to do in different situations.

#### 4.3.2 Striving for openness and a tolerant atmosphere

Patient safety is improved if healthcare professionals dare to ask each other for help. A culture that is non‐blaming, kind and that involves respect and trust for each other's professional roles was described as important “… you have to be up front with both your own knowledge and shortcomings.”

Healthcare professionals and students are encouraged to give their point of view. This openness can lead to better decisions when faced with complicated birthing situations “… that you have confidence in each other so you can speak up about what you think and can discuss a situation and demonstrate and admit that the answers aren't always obvious, that you want to discuss options.”

#### 4.3.3 Supporting new colleagues

It is important for experienced healthcare professionals to adjust their support to new colleagues to each situation and explain that it takes time to grow into a new role and to feel secure. Teaching practical skills to new colleagues was described as being relatively simple; what is more complicated is how to pass on a sense of security to the woman giving birth. Newly qualified midwives are invited by those who are more experienced to learn and receive support to increase the patient safety “… it would be good to have two midwives present at every childbirth [...] so you could watch and learn.”

### 4.4 Managing workforce and learning

The descriptive category includes two perceptions: *Having a reasonable workload* and *Learning from critical incid*ents.

#### 4.4.1 Having a reasonable workload

Having a reasonable workload is described as essential in ensuring patient safety. Workload is affected by the number of childbirths, more complicated childbirths and less experienced and lower numbers of healthcare professionals. Increasing workload requires special focus on planning and prioritizing when distributing women and tasks to healthcare professionals. Patient safety was perceived to be increased if an experienced midwife is scheduled onto each shift “… women who are difficult from a psychosocial perspective so to say, who are in a lot of pain and are demanding, might need a more competent midwife with more experience.”

When informants described an extreme workload, the risk of missing information increasing due to communication failure and difficulty in reading birth records and carrying out rounds. Extreme workload may lead to stress and a limited overview when midwives have to take care of several women simultaneously and physicians have to manage multiple interventions at the same time. Women without specific medical needs may not receive enough attention “… she had to lie there in significant pain and anxiety all alone, no one had time for her and that is not patient safety.”

#### 4.4.2 Learning from critical incidents

Reflecting on better and worse birthing situations to increase learning was described as essential, but may be difficult to achieve regularly “… if a midwife and a physician have been on a case … that perhaps resulted in something unexpected, it is really good if this knowledge and experience is shared.”

## 5 DISCUSSION

The main findings of the study show that healthcare professionals' perceptions of patient safety with a focus on the woman in connection to childbirth were identified in four descriptive categories: *Safeguarding the woman*, *Safeguarding the birth process*, *Respecting the individual and the team* and *Managing workload and learning*.

### 5.1 Safeguarding the woman

Providing supportive care and listening to the woman were perceptions of patient safety. Despite previous research has found that supporting and listening to the woman is of importance (Bradfield, Duggan, Hauck, & Kelly, 2018; WHO, 2018), the results have not been related to patient safety. Shakibazadeh et al. (2018) emphasized that listening to the woman is a critical component of respectful childbirth care. In the present study, a risk for unsafe communication in terms of language barrier was found. Support from a community‐based doula could improve communication with and transcultural care of foreign‐born women and facilitate midwives' work (Akhavan & Lundgren, 2012). Despite the emphasis on effective communication (Renfrew et al., 2014), there is a lack of evidence on how to support communication between healthcare professionals and women during childbirth (Chang, Coxon, Portela, Furuta, & Bick, 2018).

### 5.2 Safeguarding the birth process

The informants perceived that it was important to follow the birthing process closely and to be aware of possible risk conditions and avoid unnecessary interventions such as instrumental vaginal birth and caesarean section. Avoiding unnecessary interventions and thereby prevent patient harm has relevance to childbirth care (Shakibazadeh et al., 2018). From an international perspective, the Swedish rate of these mode of births is relatively low (Euro‐Peristat Project, 2018), while the rate of caesarean sections is increasing globally (Betran et al., 2018).

The perception of following the stage of labour describes the importance of not missing information by using a structured communication method for handovers between healthcare professionals. When handovers fail, information can be misinterpreted and lead to patient harm (The Joint Commission, 2017). The benefit of using a structured communication method such as Situation, Background, Assessment, Recommendation (SBAR) is a finding similar to other studies (Muller et al., 2018). Most Swedish labour wards have implemented SBAR (Löf, 2017), which is a recommended handover method (The Joint Commission, 2017). Interestingly, a finding in our study was that compliance to easily accessed guidelines was perceived to strengthened patient safety. Other studies have described guidelines as a cause of tension or as restricting individual autonomy in decision‐making (Hansson, Lundgren, Hensing, & Carlsson, 2019; Hunter & Warren, 2014; Robertson & Thomson, 2016).

### 5.3 Respecting the individual and the team

Using each other's competence was found to be important for patient safety by the informants. This has relevance to teamwork and the benefits of team training (Salas et al., 2008) leading to fewer medical mistakes being made (Manser, 2009). Studies show that midwives sometimes restricted teamwork by being dominant (Sinni, Wallace, & Cross, 2014) or causing confusion in the surrounding team (Hansson et al., 2019). When childbirths were video analysed, it was uncovered that physicians and midwives did not use each other's competence sufficiently about both technical and non‐technical skills (Kimmich, Zimmermann, & Kreft, 2018).

Striving for openness and a tolerant atmosphere that is kind, respectful and trusting identified by the informants as being connected to daring to ask for help. The value of facilitating trust and respectful communication for better decision‐making was supported by Rönnerhag, Severinsson, Haruna, and Berggren (2019) who explored patient harm during childbirth. Mutual support and open communication are important team competencies to create a positive patient safety culture (Kohn, Corrigan, & Donaldson, 2000; Nieva & Sorra, 2003).

### 5.4 Managing workforce and learning

Having a reasonable workload is an essential precondition for safe conditions in the labour ward, according to the informants' perceptions, and an extreme workload was perceived as a safety threat. Pressure connected to high workload was prominent in other studies, but not connected to patient safety (Aune, Amundsen, & Skaget Aas, 2014; Hansson et al., 2019; Hunter & Warren, 2014). On the other hand, studies show that high workload affect both healthcare professionals' working conditions and patient safety (Smeds Alenius, 2019; The National Board of Health & Welfare, 2018).

The informants in the study emphasized the importance of learning from critical incidents, but reflection on such incidents was not a regular practice. Learning lessons in the aftermath of patient harm is crucial for improving patient safety (Kohn et al., 2000; Nyfløt et al., 2018; Robertson & Thomson, 2016).

## 6 LIMITATIONS

Some limitations of the study must be acknowledged. We could not conduct a purposeful sampling, which is required in a phenomenographic study (Marton, 1981; Uljens, 1996); therefore, only those interested to participate in the study were included. The analysis process was dependent on the quality of the interviews. During the interviews, more probe questions could have been used to enrich the results and probably not all perceptions were captured in this study.

## 7 CONCLUSION

Healthcare professionals highlighted the importance of supportive care and listening to the woman to create safe conditions. They followed the birth process closely and avoided unnecessary interventions. It was important to use each other's competence and that the atmosphere was open and tolerant was a prerequisite for safe care and better decision‐making. A reasonable workload and learning from critical incidents were perceived as essential for ensuring safe care. The recommendations for clinical practice to improve patient safety are healthcare professionals' awareness of centring the woman and of team competencies.

## CONFLICT OF INTEREST

The authors declare no conflicts of interest.

## AUTHOR CONTRIBUTIONS

AS: contributed to the design of the study, carried out the data collection, analysed the data and interpreted the findings in collaboration with the other authors, and wrote the manuscript. MHL, CB and AKSB: contributed to the design of the study, analysed the data and interpreted the findings in collaboration with the other authors, supervised and critically revised the manuscript. All authors read and approved the final manuscript.
